# Impact of Pre-Ozonation during Nanofiltration of MBR Effluent

**DOI:** 10.3390/membranes12030341

**Published:** 2022-03-18

**Authors:** Zoulkifli Amadou-Yacouba, Julie Mendret, Geoffroy Lesage, François Zaviska, Stephan Brosillon

**Affiliations:** IEM (Institut Européen des Membranes), UMR 5635 (CNRS-ENSCM-UM2), Université de Montpellier, 34095 Montpellier, France; zoulkifli.amadou-yacouba@umontpellier.fr (Z.A.-Y.); geoffroy.lesage@umontpellier.fr (G.L.); francois.zaviska@umontpellier.fr (F.Z.); stephan.brosillon@umontpellier.fr (S.B.)

**Keywords:** wastewater reuse, organic matter, ozonation, nanofiltration, membrane fouling

## Abstract

This study aimed to investigate the impact of real MBR effluent pre-ozonation on nanofiltration performances. Nanofiltration experiments were separately run with non-ozonated real MBR effluent, ozonated real MBR effluent and synthetic ionic solution mimicking the ionic composition of the real MBR effluent. The specific UV absorbance and the chemical oxygen demand were monitored during ozonation of real effluent, and the mineralization rate was calculated through the quantitative analysis of dissolved organic carbon. The membrane structure was characterized using SEM on virgin and fouled membrane surfaces and after different cleaning steps. The results confirm the low effect of the ozonation process in terms of organic carbon mineralization. However, the chemical oxygen demand and the specific UV absorbance were decreased by 50% after ozonation, demonstrating the efficiency of ozonation in degrading a specific part of the organic matter fraction. A benefic effect of pre-ozonation was observed, as it limits both fouling and flux decrease. This study shows that the partial mineralization of dissolved and colloidal organic matter by ozonation could have a positive effect on inorganic scaling and decrease severe NF membrane fouling.

## 1. Introduction

A promising solution to the challenge of water shortage is to consider urban and industrial wastewaters no longer as wastes but more as renewable resources of water, nutrients and energy. One of the most challenging limiting factors to wastewater reuse is the widespread occurrence of micropollutants in different environmental compartments. To overcome this issue, membrane processes have been demonstrated to remove well micropollutants [1,2]. Among the numerous available membrane processes, nanofiltration is widely recognized for the compromise it offers in terms of selectivity and flux permeability [3,4].

Nonetheless, the fouling propensity remains a very big challenge for a widespread usage of this process [5]. In fact, the nanofiltration membranes seem particularly vulnerable to severe fouling, which constitutes their main drawback. Their propensity to both organic and inorganic fouling was demonstrated by numerous previous studies [6,7]. The reduction of membrane permeability due to fouling causes a substantial increase in operational and maintenance costs and a decrease in effluent quality and membrane lifetime. 

As a solution to fouling challenges, numerous authors have investigated the impacts of different types of pretreatment processes such as advanced oxidation. Ozonation, as pretreatment to mitigate the fouling propensity in NF process, is one of the most promising technologies [8,9,10]. Former studies have pointed out the increase in permeate flux, but very few studies have focused on fouling dynamics in the combined process of ozonation/nanofiltration [11,12]. In particular, there is a lack of data about the specific roles of inorganic salts and organic matter during pre-ozonation of real wastewater before nanofiltration. 

The aim of this study was to analyze the NF process applied to a real MBR secondary effluent. Specifically, it consisted in identifying the role of organic and inorganic matters in fouling mechanisms, monitoring the degradation rate of organic matter by an ozonation process and investigating the impact of pre-ozonation on performances of subsequent NF processes. In particular, the effect of the mineralization rate of organic matter on fouling mechanisms in NF was identified.

## 2. Materials and Methods

### 2.1. Matrix Used for the Study

#### 2.1.1. Real Secondary Effluent Matrix

An effluent from a full-scale domestic WWTP equipped with MBR, located close to Montpellier, France, was used as real matrix. The plant was designed to treat 13,000 m^3^/d of domestic wastewater. The MBR was equipped with KUBOTA Submerged Membrane Unit (SMU RW400) (KUBOTA, London, Englandflat-sheet microporous membranes made of chlorinated polyethylene (total surface of 16,240 m^2^), with an average pore size of 0.2 µm. The characteristics of the MBR permeate are presented in Table 1. SUVA254 is the specific ultraviolet absorbance. The MBR effluent was immediately stored at nearly 4 °C after sampling in order to limit the variation of the composition and re-warmed at room temperature (20 °C ± 1 °C) before conducting the experiments. 

#### 2.1.2. Synthetic Ionic Solution Matrix Composition

In order to deeply investigate the impact of organic and ionic matter on fouling mechanisms, it was chosen to conduct experiments with matrix free of organic matter. Therefore, a synthetic ionic solution was prepared in ultra-pure water imitating the ionic composition of the real MBR effluent (Table 2).

To prepare the solution with the aforementioned ions, different salts were used in the following concentrations (Table 3).

### 2.2. Nanofiltration Experiments

#### 2.2.1. Membrane Selection and Characterization

The membrane used for this study is an NF-90 polyamide membrane from DOW Filmtec. It is considered as a “tight” NF membrane with an estimated MWCO around 150 Da. Before experiments, each membrane was firstly soaked in ultrapure water to remove preservative agent and then compacted at 18 bars for at least one hour or until stability of the permeate flux was reached. Thereafter, the membranes were fully characterized in terms of pure water permeability and sodium chloride rejection, with values corresponding to 8.4 ± 1.0 L h^−1^ m^−2^ bar^−1^ and 88 ± 4%, respectively.

#### 2.2.2. Cross-Flow Nanofiltration Unit and Experimental Protocol

**Figure 1:**
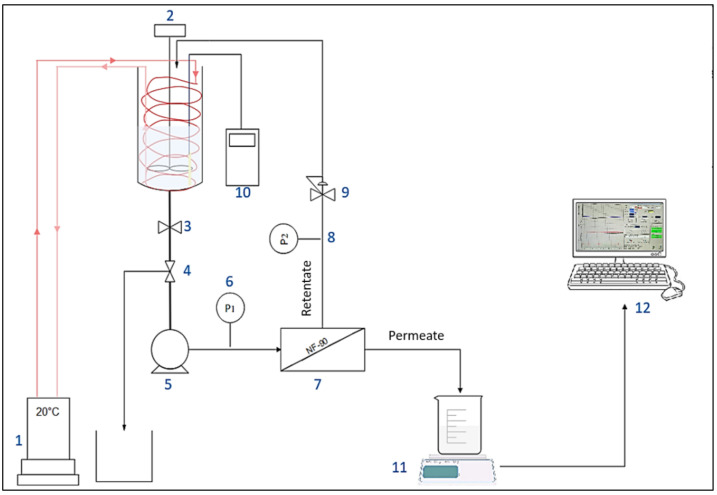
Experimental setup of nanofiltration bench-scale pilot. (**1**) Cryothermostat. (**2**) Mechanical stirrer. (**3**) Tank isolation valve. (**4**) Valve for sampling. (**5**) Pump. (**6**) And. (**8**) Pressure sensors. (**7**) Filtration unit. (**9**) Pressure control valve. (**10**) Conductivity meter. (**11**) Precision scale. (**12**) Data processing.

The filtration experiments were carried out with 140 cm^2^ flat-sheet membrane samples in an Osmonics Sepa CF II cell (Sterlitech Corp., Auburn, WA, USA). The Sepa cell was fed by a pump Hydra-Cell, Wanner Engineering, Inc, Minneapolis, MN, USA) with the solution from a 16 L feed vessel (Figure 1). The wastewater temperature was kept constant (20 ± 1 °C) using a cryothermostat (F32, Julabo, Seelbach, Germany). The bench-scale NF experiments were performed at a cross-flow velocity (vT) of 0.5 m s^−1^ with a medium foulant spacer, 47 Mil (1.194 mm). The transmembrane pressure (TMP) was set constant at 10 bars using a micrometric pressure control valve located on the retentate outlet. The membrane performances were monitored throughout the filtration experiment at ~0%, 15%, 40% and 60% until reaching 80% of water recovery (or the maximum water recovery rate reachable in case of earlier severe fouling). The flux was recorded throughout the experiment by measuring the permeate weight every 60 s. Retentate and permeate samples were collected for physico-chemical analysis. The volume of the collected sample for different analyses was considered in the apparent rejection determination. Considering that the NF system is made of stainless steel and all the tubing is in Teflon, it was assumed that compounds (organic and inorganic matter) adsorption was exclusively occurring on membrane material. 

To evaluate the impact of organic and inorganic matters on membrane fouling mechanisms, three types of NF experiments were run: (1) non-ozonated real MBR effluent, (2) synthetic ionic solution and (3) ozonated real MBR effluent.

#### 2.2.3. Membrane Fouling Propensity Test

After each filtration experiment, the NF unit was cleaned first by ultrapure water cleaning, then recirculating caustic soda (NaOH, 2%) for 6 h and finally recirculating acid solution (HNO_3_, 2%) for 6 h. After each base and acid cleaning, the system was fully rinsed with deionized water until a conductivity of 50 µS cm^−1^ and a neutral pH were reached in the NF permeate. Membrane fouling was characterized according to the flux recovery after effluent filtration and after different cleaning steps. Reversible fouling was estimated immediately after ultrapure water cleaning by comparison with water flux before the filtration, at the beginning of the experiment. Then, the irreversible fouling was determined using chemical cleaning. Two types of irreversible fouling were distinguished: organic irreversible fouling evaluated by the determination of flux recovery after NaOH cleaning and inorganic irreversible fouling (scaling) determined after acid cleaning (HCl). Flux was measured after these cleaning steps and compared to the initial flux so as to estimate the flux recovery proportion of each type of cleaning.

Membrane surface morphology, for virgin and fouled membranes and after each cleaning step, were characterized with a Scanning Electron Microscope (SEM, Hitachi Table top Microscope S-4800) interfaced with an Energy-Dispersive X-ray (EDX) spectroscopy system (Thermo-Fisher, Waltham, MA, USA). Membrane samples were coated with a thin layer of gold before SEM analysis. EDX measurements were performed at different locations on the membrane surface, in order to obtain a comprehensive elemental composition. SEM micrographs were obtained at an accelerating voltage of 2 kV and magnification of 25,000.

#### 2.2.4. Osmotic Pressure

The difference in osmotic pressure (∆π) between feed and permeate sides of the membrane was calculated using Equation (1) [13]:

(1) $$\Delta \pi =\;\pi_{\mathrm{feed}}-\;\pi_{\mathrm{perm}}$$

with π feed representing osmotic pressure in the feed side and π perm representing osmotic pressure in permeate side. 

The NF removal was high, and the ions concentrations (and consequently the induced osmotic pressure) at permeate side were negligible compared to that of feed side.

For each ion, the osmotic pressure is given by Equation (2) [13]:

(2) π =C · R · T

For all the identified ions, Equation (3) enables estimation of π [13]:

(3) $$\pi \;=R\;\cdot \;T\overset{n}{\underset{i=1}{\sum }}\mathrm{Ci}$$

with: 

R: gas constant (= 8.314 J/mol K);

T: temperature of solution (°K);

C: concentration of ion (mol/m^3^);

n: number of ions in the solution.

#### 2.2.5. Concentration Polarization

Due to concentration polarization, the osmotic pressure is not homogeneous in feed solution. In fact, the ions concentration and the induced osmotic pressure (π) are more important at membrane surface (π_memb_) than in the bulk solution (π_bulk_). These values are linked by the relation given in Equation (4) [13]:

(4) $$\pi_{\mathrm{memb}}=\;\pi_{\mathrm{bulk}}\;\cdot {\;e}^{\frac{\mathrm{Jp}}{k}}$$

with: 

k: diffusion coefficient (m^2^/s);

Jp: flux (m^3^/s/m^2^).

### 2.3. Bench-Scale Ozonation System Setup

**Figure 2:**
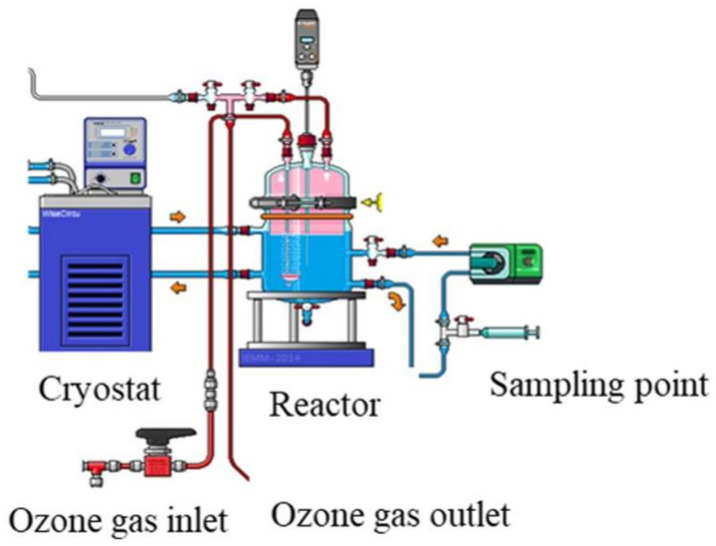
Experimental setup of ozonation bench-scale pilot.

Experiments were performed in a glass stirred batch reactor (Vreactor = 3 L) where the liquid solution is maintained at room temperature (20 °C) using a cryothermostat (Figure 2). The ozone was continuously produced from a lab-grade pure oxygen tank by an ozone generator (BMT 803 N). Before diffusion in the reactor, the ozone was diluted with oxygen at a gas flow of 60 L h^−1^ and introduced through a porous diffuser at the bottom of the reactor. The gas ozone concentration ([O_3_]gas,in) was monitored after dehumidification by an ozone gas analyzer (BMT 964). The impact of pre-ozonation on NF process was investigated for 30 min reaction contact time, and the dissolved ozone dose (TOD) was determined using indigo method [14]. 

The desired oxygen/ozone ratio was determined using two electro-valves connected to the monitoring software. The ozone dissolution rate was increased in the solution us-ing an agitator (400 rpm). The experiment consisted in applying an ozone gas concen-tration of 5 gO_3_/Nm^3^ to determine the transferred ozone dose through Equation (5).

(5) $$\mathrm{TOD}\;=\frac{\left(C_{\mathrm{ge}}-C_{\mathrm{gs}}\right)*Q_{g}*t}{V_{\mathrm{reactor}}}$$

with:

TOD: transferred ozone dose (gO_3_/m^3^);

C_ge_: gas-phase ozone inlet concentration (g/Nm^3^);

C_gs_: gas-phase ozone outlet concentration (g/Nm^3^);

Q_g_: gas flow (m^3^/h);

t: reaction time (h);

V_reactor_: reactor volume (m^3^).

Finally, the specific ozone dose [O_3_]_specific_ was calculated with Equation (6):

[O_3_]specific = TOD/TOC (6)

with:

TOC: total organic carbon (gC/m^3^)

### 2.4. Chemical Analysis

#### 2.4.1. Ionic Chromatography

The concentrations of ionic compounds were determined in all samples by ionic chromatography:

- Anionic compounds concentrations were determined with an ICS 1000 system (Thermo-Fisher, Waltham, MA, USA) equipped with a Dionex AS19 column fed by an eluent flow rate of 1 mL·min^−1^. A KOH eluent was used as mobile phase through the following gradient: 10 mM for 10 min, then 45 mM for 20 min and 10 mM for 10 min.
- Cationic compounds concentrations were determined with an ICS 900 system (Thermofisher Dionex, France) equipped with a Dionex CS12A column fed by 20 mM methanesulfonic acid at a flow rate of 1 mL·min^−1^.

#### 2.4.2. Global Indicators for Pollution Monitoring: TOC, UV254 and SUVA Analysis

The specific UV absorbance (SUVA254) corresponds to the ratio of UV absorbance at wavelength of 254 nm, measured in a 1 cm quartz cuvette using a UV–vis spectrophotometer (UV-2401PC, Shimadzu, Kyoto, Japan) and TOC value [15]. TOC analysis was performed using a TOC-VCSN Shimadzu analyzer (Shimadzu Japan).

#### 2.4.3. Scanning Electron Microscopy (SEM)

A Hitachi Microscope (Hitachi S4800 SEM) was used to inspect surfaces of the virgin and pre-fouled membranes. Small pieces were cut from the surfaces of membranes (post-mortem analysis). Before analysis, the samples were dried in desiccator until measurement in order to remove residual moisture and then metalized with platinum. The surfaces of fouled and virgin membrane were magnified 5000–15,000 times. 

## 3. Results

### 3.1. Flux Evolution and Fouling Mechanisms during Nanofiltration

**Figure 3:**
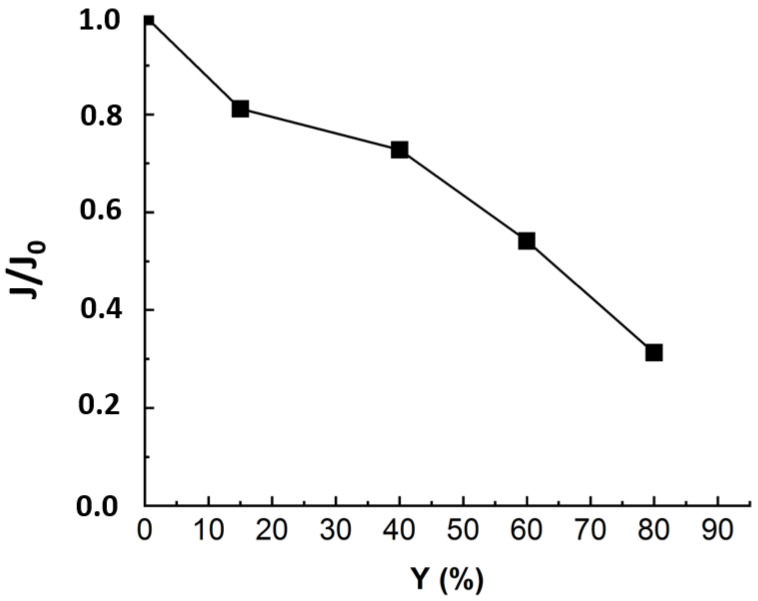
Flux evolution in NF experiment applied to MBR real effluent matrix: TMP = 10 bars, T° = 20 °C, J_0_ = 53 L·m^−2^·h^−1^, duration of the experiment = 24 h.

One of the criteria to evaluate NF efficiency is the evolution of the permeate flux with the time of filtration. The recovery rate (Y) was calculated corresponding to the ratio between the extracted permeate volume and initial feed volume. In order to compare the flux evolution for different experiments, the relative flux corresponding to the ratio between the flux at any time (J) and the initial flux (J0) was considered. Figure 3 presents the normalized flux (J/J0) during nanofiltration of real MBR effluent under a TMP of 10 bars.

Figure 3 revealed a drop of almost 70% in the initial flux value when reaching the maximum conversion rate of 80%. As established in previous studies, the main fouling mechanism during MBR effluent filtration by NF is organic fouling [11]. According to some authors, the reason that could explain the flux drop is that the organic matters, particularly those with higher MW and hydrophobicity, corresponding to humic-like substances, deposited into the pores and onto the membrane [16,17,18]. The deposited organics enhance gel layer formation, which was related to the rapid flux decline at the first stage. Then, the slower flux decrease could come from gel layer compaction and interactions between inorganic salts and organic matter deposited on the membrane surface [19]. For instance, Lin et al. have studied the roles of organic, inorganic and biological fouling along with NF applied to raw effluent. The authors noticed that organic/inorganic binary fouling became dominant, contributing up to 39.7% of flux decline due to metal/organic complexation [7]. The third stage, corresponding to a more pronounced flux drop, could come from concentration polarization [5,17]. Nonetheless, to establish a clear distinction between the impacts of organic and inorganic contributions to flux decline, it is required to run NF experiments with OM-free matrix.

### 3.2. Influence of Ionic Matrix during Nanofiltration

**Figure 4:**
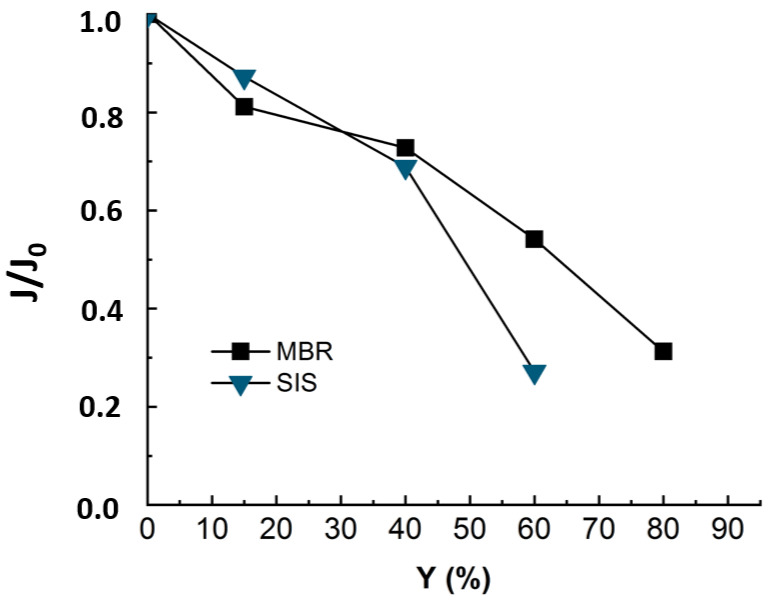
Flux evolution during NF experiment applied to MBR real effluent (duration of the experiment = 24 h) and synthetic ionic solution matrixes SIS (duration of the experiment = 18 h). TMP = 10 bars, T° = 20 °C, J_0_-SIS = 64 L·m^−2^·h^−1^, J_0_-MBR = 53 L·m^−2^·h^−1^.

To evaluate the impact of organic matter on fouling, experiments were run with synthetic ionic solution (SIS) mimicking the ionic composition of the real MBR effluent. The flux was monitored along with permeate recovery rate and is presented in Figure 4 with that of real MBR effluent matrix.

The Figure 4 revealed a decline of 75% in permeate flux at 60% of recovery for SIS solution. The occurrence of the severe fouling may be linked to an inner fouling caused by ionic compounds. In fact, as the organic matter playing the role of competitor in ions adsorption is no longer present in solution, the ions are free to adsorb onto membrane surfaces and enhance membrane fouling while diffusing through membrane pores. Thus, it was not possible to reach such high conversion rates as with real effluent (Y = 60% instead of 80%). Teixeira and Rosa have studied the impact of the water inorganic matrix on the permeate flux and the natural organic matter (NOM) removal by nanofiltration [20]. They noticed a decrease in flux in the presence of calcium. According to the authors, the flux and rejection decreased further in the presence of 1 mM Ca^2+^, which reduced the membrane negative charge and sieving effects and increased chemical interactions. In fact, in the present study, all the detected ionic composition was mimicked by a synthetic ionic solution free of OM that could compete with the membrane in adsorbing the inorganic and mitigate the inorganic fouling.

#### 3.2.1. Influence of the Osmotic Pressure

**Figure 5:**
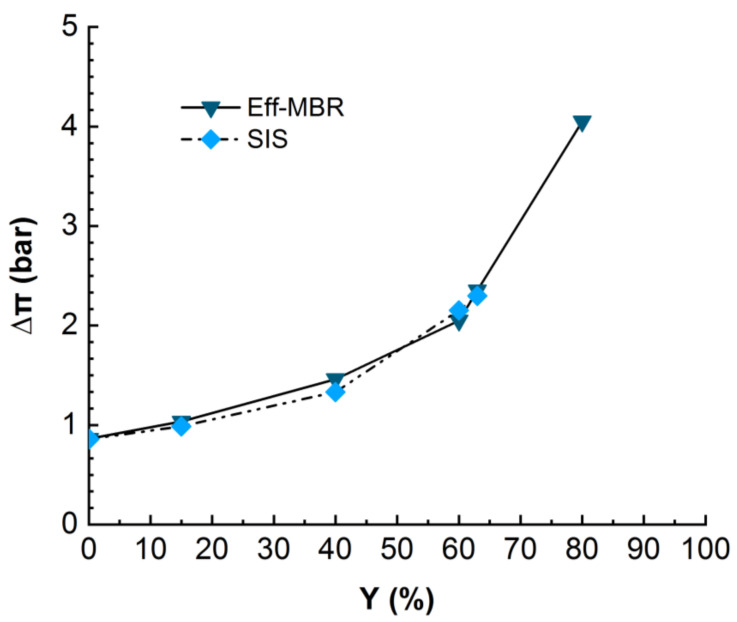
Evolution of differential osmotic pressure in NF for real MBR effluent (duration of the experiment = 24 h) and SIS (duration of the experiment = 18 h). TMP = 10 bars, T° = 20 °C.

During the nanofiltration experiments, the ionic compounds became more and more concentrated and induced an osmotic pressure, which is supposed to increase with permeate recovery. The osmotic pressure constitutes a resistance to physical pressure and should be overcome in order to get permeate flux through the membrane. The differential osmotic pressure between retentate and permeate streams was calculated for real MBR effluent and synthetic ionic solution and compared in Figure 5. 

The monitoring of the osmotic pressure revealed that it increases with permeate recovery rate from around 1 bar at the beginning to 2.2 bars at 60% of recovery rate for SIS solution and up to 4 bars for the MBR effluent matrix. The Figure 5 clearly displays a similarity in the evolution of osmotic pressure for both real MBR effluent and the synthetic ionic solution mimicking the MBR ionic composition, even though the permeate flux drastically dropped in the case of SIS much earlier than in the MBR effluent case (Figure 4). This result confirms the suspected inner fouling due to inorganic scaling. As the solution is free of OM, which would adsorb the ions, they are free to interact with each other and with the membrane, enhancing the scaling [21,22].

#### 3.2.2. Characterization of Membrane Fouling

**Figure 6:**
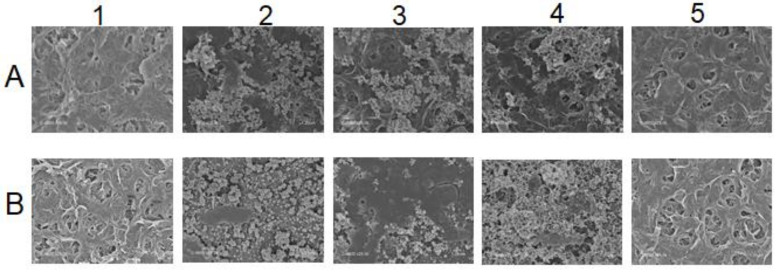
SEM of membrane surfaces at different states: (**A**) For membrane used in MBR effluent experiment, (**B**) Membrane used in SIS experiment. 1. Virgin membrane, 2. Fouled after experiment, 3. UPW-cleaned membrane, 4. Base-cleaned and 5. Acid-cleaned.

At the end of each experiment, the membrane goes through different cleaning steps beginning with ultrapure water, followed by basic and acid-based cleanings, respectively. Scanning Electron Microscopy (SEM) analysis was applied to samples from membrane used for both real MBR effluent and SIS. Samples of virgin and fouled membrane and membrane after the different cleaning steps were used, and the results are presented in Figure 6.

Figure 6 visually illustrates the membrane surface state throughout the different steps. A mixture of inorganic and dissolved organic matter can be noticed on the fouled membrane used with the real MBR effluent (2.A), while the membrane fouled with SIS (2.B) displays disaggregated inorganic compounds only. The subsequent cleaning methods helped to identify the type of fouling that occurred during these experiments through foulants characterization [16,23]. In fact, for the membrane fouled by real MBR effluent, while the ultrapure-water-based cleaning likely removed part of the fouling matter (3.A), the sodium hydroxide cleaning significantly removed it, except for some inorganics (4.A) that were totally removed by hydrogen chloride acid washing (5.A). For the SIS-fouled membrane, on the other hand, the ultrapure-water-based cleaning was able to remove part of scaling (3.B). The sodium hydroxide cleaning was not able to remove the inorganics on the membrane surface (4.B). Only the acid cleaning totally recovered the fouled membrane surface to almost virgin state (5.B).

### 3.3. Influence of Pre-Ozonation during Nanofiltration of Real MBR Effluent

#### 3.3.1. Monitoring of Organic Matter

**Figure 7:**
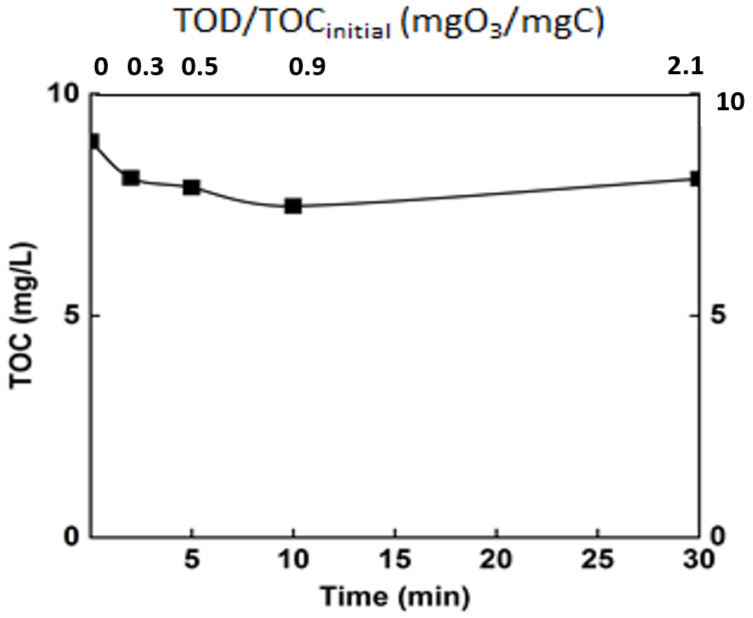
Monitoring of the mineralization rate of the real matrix during ozonation. T° = 20 °C, Vreactor = 3 L, Vstir = 400 rpm, [O_3_]gas = 5 gO_3_/Nm^3.^

To evaluate the impact of organic matter and its degradation by ozone on the performances of nanofiltration process, the mineralization rate of organic matter was monitored during ozonation process, and the results are given in Figure 7.

**Figure 8:**
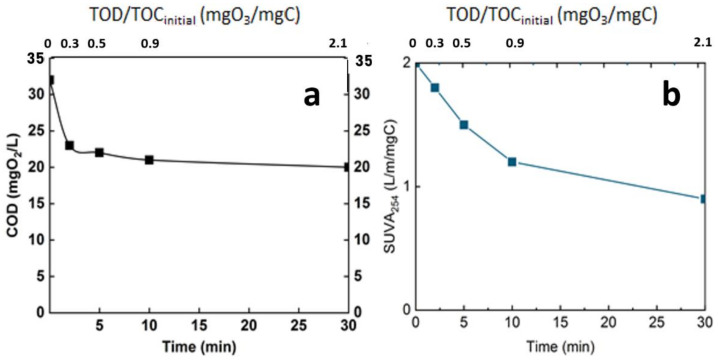
Evolution of global parameters of the real matrix during ozonation. (**a**): COD, (**b**): SUVA254, T° = 20 °C, Vreactor = 3 L, Vstir = 400 rpm, [O_3_]gas = 5 gO_3_/Nm^3^.

Ozonation, as revealed by some previous studies, is not sufficient to completely degrade organic matter [8]. This is confirmed by the current study, in which only a mineralization of 15% was achieved after 30 min (TOC around 8 mg/L). Even though the mineralization rate was relatively low, the ozonation engendered an important change in the organic matter. Indeed, even if the mineralization of the organic matter was moderate, chemical changes occur, and the efficiency of ozonation in terms of modification of organic matter structure was monitored through some common parameters. The chemical oxygen demand and the specific UV absorbance (SUVA254) are some of these indicators (Figure 8).

Both of the two parameters indicate the efficiency of ozonation process in oxidizing organic matter. The COD decreased from 33 mgO_2_/L to 23 mgO_2_/L after 3 min reaction time and to less than 20 mgO_2_/L at 30 min of reaction time (Figure 8a), corresponding to the introduction of oxygen in the chemical structure of the organic matter. This level of mineralization was already observed by Gong et al. and Justo et al. [15,24]. In addition, the ozonation decreased the SUVA by half after 30 min reaction time; this indicates the opening of the double bond mainly in the aromatic group. This parameter is a good indicator of the change in the chemical structure of the organic matter [25,26] (Figure 8b).

#### 3.3.2. Nanofiltration of Ozonated Real MBR Effluent

**Figure 9:**
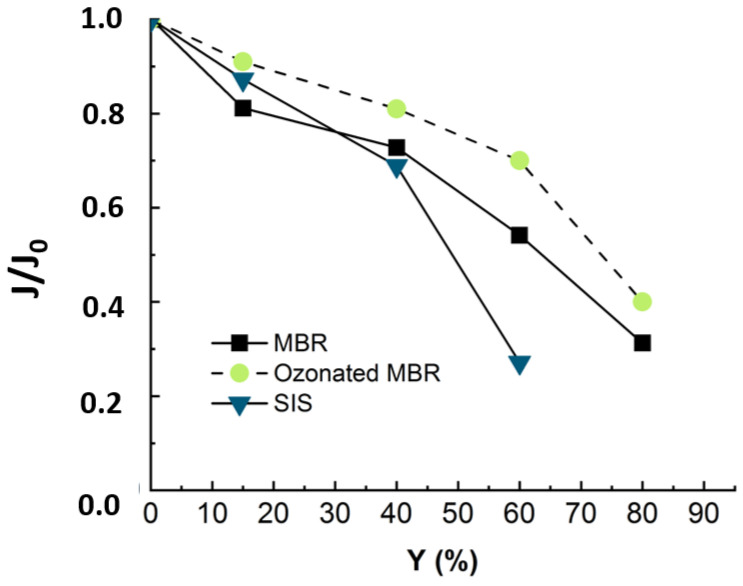
Flux evolution in NF experiment applied to synthetic ionic solution (duration of the experiment = 18 h) and non-ozonated (duration of the experiment = 24 h) and ozonated MBR real effluent matrixes (duration of the experiment = 24 h). Ozonation reaction time = 30 min, T° = 20 °C, Vreactor = 3 L, Vstir = 400 rpm, [O_3_]gas = 5 gO_3_/Nm^3^ TMP = 10 bars, J_0_-MBR = 53 L·m^−2^·h^−1^, J_0_-MBR + O_3_ = 54 L·m^−2^·h^−1^, J_0_-SIS = 64 L·m^−2^·h^−1^.

During the NF experiment applied to the ozonated real MBR effluent, the flux evolu-tion was monitored, and the relative flux is displayed in Figure 9 in comparison with non-ozonated real MBR and SIS matrix.

Figure 9 reveals that when the nanofiltration experiment is run with ozonated real MBR effluent, the drop in flux trends is slightly slower, as around 10% of flux was re-covered by pre-ozonation. Even though the ozonation is not efficient in terms of mineralization, it changes the structure of the organic matter [12]. According to the cited authors, pre-ozonation increases the hydrophilic fraction and anionic charge of organics and alters their size distribution [1,12]. In fact, the gel layer (coming from organic and inorganic complexation) was demonstrated to be responsible for membrane fouling. Therefore, the ozonation, by degrading part of this gel layer, leads to improve nanofiltration conditions by reducing the fouling celerity [11].

When the NF experiment was applied to non-ozonated real MBR effluent, the fouling essentially came from complexation of organic and inorganic matter [11]. When the nanofiltration experiment was run with SIS free of organic matter, the drop in flux trends was much more severe and occurred earlier (Figure 9). This demonstrated that during NF experiments, the propensity to inorganic fouling is much higher than that of organic fouling. After ozonating the real MBR, the trend of permeate flux in NF is improved due to delayed fouling, as the ozonation was insufficient to totally mineralize the effluent organic matter. These results demonstrate that the main drawback of the ozonation process, which is its limited mineralization rate, rather constitutes an advantage for a subsequent NF process: the residual organic matter prevents a severe inorganic fouling by competing with the membrane for adsorption of inorganics. For instance, Li et al. have studied the operational optimization and membrane fouling analysis of nanofiltration in municipal wastewater advanced treatment [16]. One of the main conclusions they came to is that inorganic fouling was mitigated because the inorganics were assumed to adsorb on the effluent organic matter.

#### 3.3.3. Cleaning and Nanofiltration Performances Recovery

Two other parameters used to characterize the fouling that occurred during NF experiments are the type of cleaning and the rate of flux that it allowed to be recovered. The values of flux at the beginning and the end of the experiments are recapitulated for all matrixes in Table 4.

**Figure 10:**
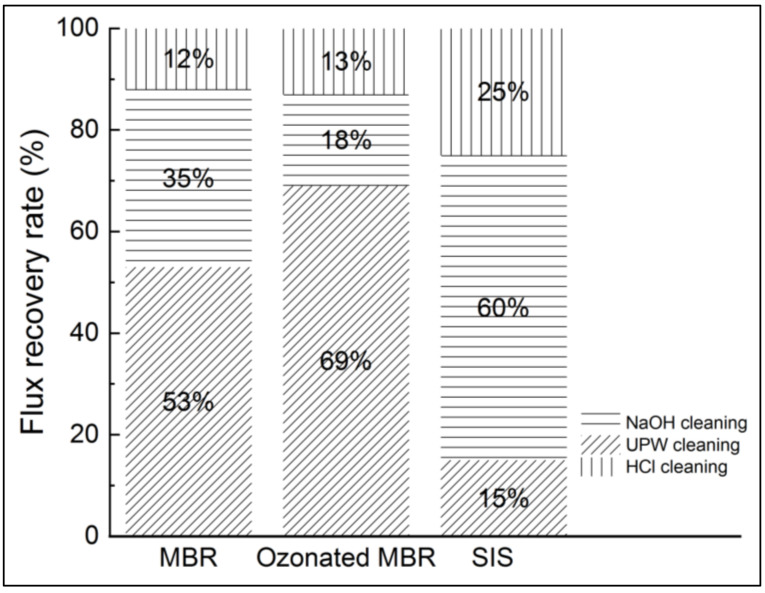
Flux recovery after pure water, NaOH and HCl cleanings in NF experiments applied to non-ozonated and ozonated real MBR effluent and synthetic ionic solution.

Hence, after each nanofiltration experiment, ultrapure water was used to clean the membrane, and the permeability was measured. A basic cleaning using NaOH (0.1 N) and acid cleaning using HCl (0.1 N) were successively performed as well. It consisted in imbibing the membrane in the cleaning solution for 6 h for both chemical solutions. The membrane permeability recovery rates were determined for the three studied matrixes and are presented in Figure 10.

According to Figure 9, the SIS induced more severe fouling than the real MBR effluent. Then, ultrapure water cleaning enabled 53%, 69% and 15% flux recovery for MBR effluent, ozonated MBR effluent and SIS solution, respectively. Fouling corresponding to both MBR effluent and ozonated MBR effluent thus seem easier to remove, which is consistent with Figure 9. Sodium-hydroxide-based cleanings allowed non-negligible permeate flux recovery in the cases of real MBR effluent (35%) and ozonated MBR effluent (18%) and a significant flux recovery of SIS-fouled membrane (60%), for which chemicals are needed. The acid-based cleaning allowed the most important recovery for SIS solution, which is consistent with the inorganic nature of fouling in this case. According to Li et al., the water flushing samples after nanofiltration of wastewater were essentially composed of low MW with high intensity, which is typically related to humic substances, indicating that the humic substances could be removed easily by physical cleaning, which is in accordance with the present result during ultrapure water cleaning [16]. In the case of severe inorganic fouling, the acid-based cleaning is required for flux recovery [17,23,27].

## 4. Conclusions

This research aimed to evaluate the impact of pre-ozonation on fouling propensity in nanofiltration. The fouling was mainly due to organics and inorganics complexation forming a gel layer (70% drop in flux at 80% of permeate recovery). When the NF experiment was run with an organics-free synthetic ionic solution, the fouling was more severe because of the high propensity of NF to inorganic fouling (75% drop in flux at 60% of permeate recovery). When the ozonated real MBR effluent was used for NF experiment, not only was the fouling delayed (62% drop in flux at 80% of permeate recovery), but the flux recovery was improved as well by a mere water cleaning. Therefore, pre-ozonating the effluent presents two advantages: it allows economical use of chemicals needed for chemical cleaning, and it contributes to improving the membrane lifetime by delaying chemical cleaning.

The SEM analysis confirmed that the acid cleaning was the most efficient to recover a virgin membrane state, even though the ultrapure water and basic cleanings can allow recovering an important part of flux, depending on the type of fouling linked to the nature of matrix used for the experiment.

The results demonstrate that the low mineralization rate of ozonation process is of high value to preventing a severe inorganic fouling. It mitigates the organic fouling by degrading partially and modifying the molecular structures of organic matter, which improves its hydrophilicity. On the other hand, the remaining organic matter, which resulted from the partial mineralization, prevented the membrane from a severe fouling, as a total mineralization would lead to occurrence of inorganic scaling. To sum up, ozonation might be the best AOP to couple with an NF process for better organic and inorganic fouling mitigation for wastewater reuse.

## Figures and Tables

**Table 1:** Characteristics of real MBR effluent (*n* = 5).

Parameters	Unit	Average	Minimum	Maximum
pH		7.40	7.10	7.80
Electric conductivity	µS/cm	3300	2460	3940
TOC	mgC/L	6.70	5.50	8.60
COD	mg O_2_/L	19.10	13.60	23.00
Absorbance at 254 nm		0.14	0.13	0.16
SUVA_254_	L/mg/m	2.1	1.9	2.4
TSS	mg/L	2.50	2.30	2.70

**Table 2:** Ionic composition of real MBR effluent and synthetic ionic solution.

Parameters	Unit	Real MBR Effluent	Synthetic Ionic Solution	Diffusion Coefficient k (m^2^/s)
Ammonium NH_4_^+^	mg/L	1.80	2.00	0.51
Bromide Br^−^	mg/L	1.20	0.00	1.46
Calcium Ca^2+^	mg/L	134.70	130.70	0.58
Chloride Cl^−^	mg/L	602.10	640.30	1.47
Hydrogen carbonate HCO_3_^−^	mg/L	254.00	290.50	1.00
Magnesium Mg^2+^	mg/L	48.50	47.50	0.51
Nitrate NO_3_^−^	mg/L	9.00	7.30	1.38
Nitrite NO_2_^−^	mg/L	7.70	0.00	1.39
Orthophosphate PO_4_^3−^	mg/L	10.00	9.60	0.44
Potassium K^+^	mg/L	34.10	30.40	1.42
Sodium Na^+^	mg/L	321.90	324.10	0.96
Sulfate SO_4_^2−^	mg/L	153.70	101.40	0.78

**Table 3:** Salts used to prepare the synthetic ionic solution.

Compounds	Concentration (mg/L)
NaCl	400
CaCl_2_, 2H_2_O	477
MgCl_2_, 6H_2_0	400
Na_2_HPO_4_, 2H_2_O	18
Na_2_SO_4_	150
NaHCO_3_	400
KCl	60
NaNO_3_	10
NH_4_Cl	2

**Table 4:** Values of permeate flux at the beginning and end of studied NF experiments.

Matrixes Used for NF Experiments	Unit	Flux at the Beginning of the Filtration Experiment	Flux at the End of the Filtration Experiment
Non-ozonated MBR effluent	L·m^−2^·h^−1^	53	17
SIS	L·m^−2^·h^−1^	64	17
Ozonated MBR effluent	L·m^−2^·h^−1^	54	21

## Abbreviations

AOP	Advanced Oxidation Process
COD	Chemical Oxygen Demand (gO_2_/m^3^)
Da	Dalton
DCOM	Dissolved and Colloidal Organic Matter
DWW	Domestic Wastewater
EDX	Energy-Dispersive X-ray
MBR	Membrane Bioreactor
MWCO	Molecular Weight Cut-Off
NF	Nanofiltration
NOM	Natural Organic Matter
OM	Organic Matter
[O_3_]gas	Applied gas ozone concentration (gO_3_/Nm^3^)
SEM	Scanning Electron Microscopy
SIS	Synthetic Ionic Solution
T°	Temperature
TMP	Transmembrane Pressure (bar)
TOC	Total Organic Carbon
TOD	Transferred Ozone Dose
TSS	Total Suspended Solid (mg/L)
UPW	Ultrapure Water
UV254	UV absorbance at 254 nm of wavelength
v	Cross-flow velocity (m/s)
Vreactor	Volume of reactor (m3)
Vstir	Stirring velocity (rpm)
WWTP	Wastewater Treatment Plant
Y	Permeate recovery rate (%)
